# Validity and reliability of upper body push and pull tests to determine one-repetition maximum

**DOI:** 10.1371/journal.pone.0288649

**Published:** 2023-07-13

**Authors:** Eirik Sigvaldsen, Irineu Loturco, Fredrik Larsen, Jo Bruusgaard, John Magne Kalhovde, Thomas Haugen

**Affiliations:** 1 School of Health Sciences, Kristiania University College, Oslo, Norway; 2 NAR—Nucleus of High Performance in Sport, São Paulo, Brazil; Universitatea de Medicina si Farmacie Victor Babes din Timisoara, ROMANIA

## Abstract

**Objectives:**

The purpose of this study was to explore the validity and reliability of three different strength testing approaches to determine one-repetition maximum (1RM) in the bench press and prone bench pull.

**Methods:**

Twenty-eight recreationally active subjects (25 ± 2 years, 178 ± 8 cm, 78 ± 9 kg) were assessed for load-velocity (L-V) relationship, 1RM, maximal isometric force (MIF), and maximal repetitions to failure (MRF) in a Smith Machine on three separated sessions. Linear regression was used for L-V relationship, MIF, and MRF to predict 1RM. Level of significance was set to ρ ≤ 0.05.

**Results:**

Reliability analyses of the varying 1RM estimations revealed mean differences from 0.6 to -1.3 kg (mainly trivial effects) between test days 1 and 2, intraclass correlation coefficient was > 0.96, and coefficient of variation (CV) was in the range 2.3–8.3% for all tests. Regarding validity, all 1RM predictions exhibited a mean difference ≤ 1.3 kg (trivial), except for the L-V relationship method that underestimated the predicted 1RM by 5 kg (small) compared to the actual bench press 1RM. However, the L-V relationship method showed the least mean absolute errors. CVs were in the range 4.5–13.2%. Standard error of the estimate was in the range 3.2–9.7 kg. Change scores for all tests were significantly correlated with change scores in actual 1RM, except for MIF in the prone bench pull. Smallest deviations in 1RM predictions were observed for the L-V relationship approach.

**Conclusions:**

All 1RM prediction methods were highly comparable to the traditional 1RM test. However, given the high variability associated with individual predictions for each method, they cannot be used interchangeably.

## Introduction

Muscular strength is a crucial determinant for varying measures of motor performance, including speed, agility, power, balance, coordination, flexibility, jumping and throwing ability [1–3]. Well-developed strength is also important for health, and daily-life well-being [1, 4, 5], as regular doses of strength training have been shown to decrease premature mortality, delay muscular atrophy processes, prevent osteoporosis, and reduce the risk of coronary heart disease and non-insulin-dependent diabetes [1, 2, 4].

Strength capacity assessments are an integrated part of the training process to evaluate conditioning programs and analyze individual training and health status [6]. The most widely used practical method to measure dynamic muscular strength is the one-repetition maximum (1RM) test [6, 7]. 1RM refers to the heaviest weight a person can lift with maximum effort in a single repetition while maintaining the correct lifting technique [7, 8]. Other strength test methods include maximal repetitions to failure (MRF) (i.e., perform as many repetitions as possible at a certain absolute weight or a given percentage of body weight) [9–11] and maximal isometric force (MIF) [12–14]. Submaximal tests have also been applied in clinical or athletic settings to estimate 1RM indirectly, including the load-velocity (L-V) relationship method (i.e., subjects typically perform 2–10 repetitions of gradually increasing resistance loading with maximal velocity) [15–17]. The latter approach is considered feasible for sedentary and elderly persons, but also for athletes across varying time points of the season, as most maximal strength tests are accompanied with larger degree of fatigue and subsequent need for recovery, in turn upsetting the subsequent training practices or competitions negatively.

There is limited information available regarding the relationship and agreement among varying upper body push and pull tests to determine 1RM. Garcìa-Ramos et al. [18] observed that MRF at 83 ± 4% of 1RM overestimated the actual 1RM, while the L-V relationship model proposed by Sánchez-Medina et al. [19] underestimated the 1RM when performing prone bench pull with free weights. The authors in the latter study concluded that the individual L-V relationship was the most accurate method for predicting 1RM during the free-weight prone bench pull exercise. However, the athlete sample consisted mainly of well-trained rowers, and more research is needed to explore these relationships among recreationally active subjects. Moreover, Fernandes et al. [20] reported moderate to high absolute errors for varying prediction models in bench press when studying young and middle-aged resistance-trained males. Strong correlations between maximal dynamic strength and isometric force production have been observed [21, 22], but it remains unclear whether MIF provides more accurate estimates of the 1RM than other methods. Furthermore, no studies to date have investigated change score relationships among actual and predicted 1RM estimations.

The purpose of this study was to gain more insight regarding the relationships and agreement among commonly used upper-body tests and their ability to determine maximal strength in recreationally active subjects. We have therefore investigated the validity and reliability of commonly used tests to determine maximal strength in the bench press and prone bench pull, using the actual 1RM value as reference. We also aimed to evaluate the tests’ ability to track training-related changes. This will serve as useful background information when designing test batteries for health examination purposes or performance analyses.

## Materials and methods

### Participants

Twenty-eight recreationally active participants (twenty-two men and six women, 25 ± 2 years, 178 ± 8 cm, 78 ± 9 kg) volunteered to take part in this study. They were recruited in October-December 2021, and the data collection was undertaken in January-March 2022. All participants were healthy and free from injuries during the testing, and previous experience with strength training ranged from six months to 10 years. They received written information regarding the benefits and risks of the study prior to signing an institutionally approved informed written consent document (as outlined by the Norwegian Centre for Research Data) to participate in the research project. Parental consent was not necessary, as all participants were >18 y. The Regional Committee for Medical and Health Research Ethics waived the requirement for ethical approval for this study. The ethics of the project was performed according to the institutional requirements at the School of Health Sciences, Kristiania University College. Approval for data security and handling was obtained from the Norwegian Centre for Research Data (reference number 156267).

### Experimental design

**Fig 1:**
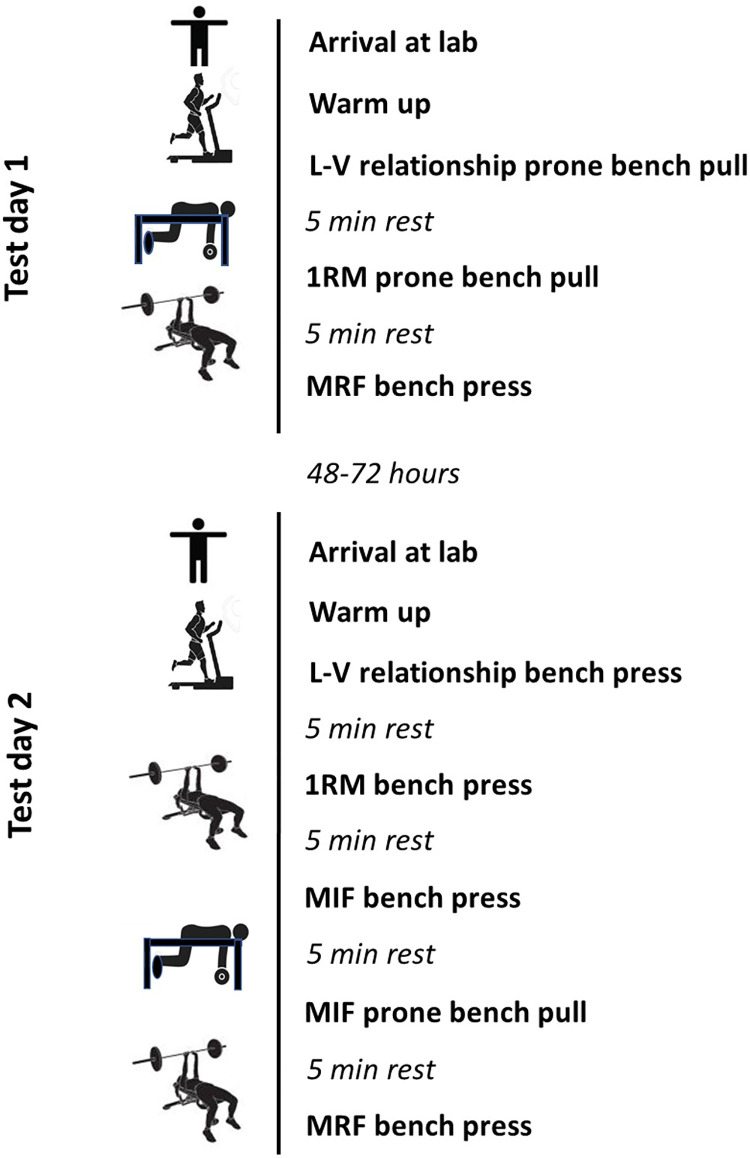
Flowchart of test session protocol. L-V = load-velocity, MIF = maximal isometric force, MRF = maximal repetitions to failure.

All subjects underwent three test sessions (session 1, 2 and 3), and each test session was performed over two days (test day 1 and 2) with 48–72 hours in between. Fig 1 shows a flowchart of the entire test session protocol. Test day 1 consisted of L-V profile in the prone bench pull, followed by direct 1RM testing in the prone bench pull, and finally MRF in the bench press. Test day 2 consisted of L-V profile in the bench press, direct 1RM assessment in the bench press, MIF in bench press, MIF in prone bench pull, and finally MRF in the prone bench pull. Five minutes recovery between each test were provided for all test days. The testing procedure was based on rigorous pilot testing. Session 1 and 2 were separated by 1–2 weeks, and the subjects were encouraged to avoid heavy upper-body strength training in this period. The test results from sessions 1 and 2 formed basis for reliability calculations. Sessions 2 and 3 were separated by 8 ± 2 weeks (mean ± SD) to assess training-related changes, and the data from session 3 were used as criterion for validity analyses.

### Procedures

Each subject performed all test sessions at the same time of day and was instructed to use identical clothing and footwear in all sessions. They were also instructed to avoid all form of high intensity training the last 48 hours prior to each test day. All tests were performed in a Smith machine rack (Eleiko, Halmstad, Sweden). The subjects were given a brief introduction regarding technique and execution prior to each exercise, and a test leader supervised all lifts. A standard warm-up procedure was performed on each test day, consisting of dynamic stretching, bodyweight exercises and 2–3 sets x 4–6 repetitions in the specific exercises with easy to moderate resistance loading (i.e., up to ~80% of expected 1RM). Recovery time between each test was set to 5 min.

In the prone bench pull, subjects laid in a prone position with the chin on the padded edge of a high bench. In the starting position, the barbell was grasped with hands slightly wider (~5 cm) than shoulder width and arms fully extended. From this position, the weights were pulled upwards with leading elbows until the barbell struck the underside of the bench and thereafter lowered to the starting position. The thickness of the bench was 9 cm. The subjects had to keep their legs and chest on the bench during the entire lift for the repetition to be approved.

In the bench press, the subjects laid on their back with eyes under the bar and feet positioned flat on the floor. Hand grip was approximately 1.5 x shoulder with. The barbell was removed from the rack, lowered, paused briefly on the chest, and then returned to fully extended arms and elbows locked. Head, shoulders, and buttocks had to be in contact with the bench surface during the entire lift, and no bouncing on the chest was allowed. Powerlifting suit was prohibited, but a belt was allowed during testing. A spotter was placed behind the athletes for safety reasons during the heaviest lifts.

#### L-V profile tests

During the L-V profile tests, all subjects were tested across increasing loads with 30 s rest in between. The subjects started with the 20 kg barbell (Eleiko, Halmstad, Sweden) only, and the resistance loading increased 5 kg for women and 10 kg for men thereafter. The weakest subjects managed to perform 6–7 incremental steps, while most subjects stopped testing once 10 incremental steps were completed. The first steps felt easy for most subjects, while the last 1–2 steps were perceived heavy, except for the strongest subjects. The subjects were instructed to lift the barbell as fast as possible during each repetition. Two attempts per resistance loading were provided (except for the resistance loadings close to 1RM), but additional trials were performed in some cases when an attempt was not approved by the test leader. Best trial (in terms of highest velocity) for each resistance load was retained for analysis. Barbell velocity was assessed by an encoder (GymAware, Kinetic Performance Technology, Australia). This device has recently been assessed for validity and reliability [23]. The encoder was attached to the barbell on the outside of the weight plates. Estimation of 1RM from the L-V relationship test was based on a linear regression model, plotting the inverse-linear relationship between mean concentric velocity and absolute loads across the incremental loading test. Terminal velocity (TV) of 0.2 and 0.5 m·s^-1^ were used for the bench press and prone bench pull, and these values were derived from preliminary 1RM pilot testing.

#### 1RM tests

Starting loads for the 1RM tests were individually determined based on experiences from preceding warm up and/or L-V testing, and the aim was to start at approximately 90% of the expected 1RM. After each successful repetition, 1–10 kg resistance loading was added (depending on performance level and perceived resistance during the previous repetition) until maximal resistance/failure was achieved. Each subject performed three to four repetitions with ~2 min recovery in between. Best performance approved by the test leader was retained for analysis.

#### MIF

During the isometric strength tests, two force platforms (FDMax, Vald Performance, Brisbane Australia) were placed under the bench, one behind and one in front. The bar height was individually adjusted to perform the prone bench pull with arms fully extended and 45° elbow angle during the bench press. The same individual bar height was used across all tests. All subjects performed 3–4 specific warm-up trials with gradually increasing effort (50–80% of maximal perceived effort). Immediately prior to each test trial, the subjects were instructed to relax in the starting position (no pre-tensioning), but at the same time ensure that all slack in the involved joints was removed. After a verbal start signal was given by the test leader, the subjects were encouraged to pull or push with maximal effort for 4–5 s until a stop signal was given by the same test leader. Up to three attempts were provided. If the maximum force increased from the first to the second trial, a third attempt was performed. ForceDecks version 1.7.0 (Vald Performance, Brisbane, Australia) was used to calculate MIF. Linear regression was applied to predict 1RM from the maximal isometric tests. Here, MIF test results were scatter plotted against the corresponding 1RM test results, and the best fit linear equation (Y = a + bX, where Y denotes the predicted 1RM, a denotes the Y intercept, b denotes the slope of the line, and X denotes the MIF test result) was used to predict the 1RM value. For simplicity, the equations derived from the third testing session formed bases for all calculations.

#### MRF tests

Based on experiences from preliminary pilot testing of recreationally active adults, the resistance loading during MRF was set to 40 and 50% of bodyweight for women and men, respectively. These loadings ensured that even the weakest participants managed to achieve some repetitions. The subjects were instructed to perform as many repetitions as possible until task failure/muscular exhaustion, and lifting tempo was self-determined. No recovery periods between repetitions were allowed. As for the MIF tests, linear regression analyses of third test session data were performed to predict 1RM from the MRF test, using 1RM as a dependent variable and number of repetition and absolute resistance as predictors.

### Statistical analyses

Excel 2019 (Microsoft; Redmond, WA, USA), SPSS 28 (IBM, Armonk, NY, USA) and GraphPad Prism 9 (Boston, MA, USA) were used for statistical analyses. A Shapiro-Wilk test was applied to check for normal distribution. A Wilcoxon-test was performed in addition to a t-test in cases where test-retest reliability data were not normally distributed, yielding equal results. Other reliability measures included the intraclass correlation coefficient (ICC), effect size (ES), standard error of the measurement (SEm), and coefficient of variation (CV%). Validity measures of the prediction methods included Pearson’s correlation (r), mean absolute error (MAE), standard error of the estimate (SEE), and CV%. The strength of the correlations was determined using the following criteria: trivial (<0.1), small (0.1–0.3), moderate (0.3–0.5), high (0.5–0.7), very high (0.7–0.9), or practically perfect (>0.9) [23]. A paired-samples T-test was performed to determine differences between the 1RM predictions and the 1RM. Brown-Forsythe and Welch’s one-way ANOVA with Dunn’s multiple comparisons tests were used to compare validity of 1RM estimates across test methods. Linear regression with Extra Sum-of-squares F-test was used to compare differences in slopes. The level of significance was set to ρ ≤ 0.05. Effect size (Cohen’s d) was used to calculate differences in means, and the following thresholds were used to express the magnitudes: trivial <0.2, small 0.2–0.59, moderate 0.60–1.19, large 1.2–1.99, or very large >2.0 [24]. Confidence intervals were set to 95% for both reliability and validity analyses.

## Results

### Reliability

Reliability values for the assessed tests are shown in Table 1. For the bench press, significant differences between test sessions 1 and 2 were observed for all methods, except for the L-V relationship. However, all these between-session differences were trivial (Table 1). All CV%-values were in the range 2–3%, except for MIF (8.3%). All ICCs were nearly perfect (≥ 0.96), and SEm values were in the range 1.2–1.6 kg for all methods, except for MIF (4.2 kg).

**Table 1:** Between-session reliability among the analyzed strength test methods.

*Bench press*	Session 1 (kg)	Session 2 (kg)	Mean diff. (kg)	p	ES (95%CI)	CV % (95%CI)	ICC (95%CI)	SEm
1RM	78.3 ± 27.8	79.5 ± 27.3	-1.2	<0.01	0.05 (-0.42, 0.33)	2.3 (1.7, 2.9)	1.0 (0.99, 1.0)	1.2
L-V relationship	75.5 ± 21.0	76.2 ± 21.7	-0.7	0.12	-0.03 (-0.41, 0.35)	2.8 (2.1, 3.6)	1.0 (0.99, 1.0)	1.4
MIF	76.2 ± 27.7	79.4 ± 25.3	-3.2	0.02	-0.12 (-0.49, 0.25)	8.3 (6.1, 10.5)	0.96 (0.91, 0.99)	4.2
MRF	78.2 ± 26.3	79.5 ± 25.9	-1.3	0.01	-0.05 (-0.42, 0.32)	3.0 (2.2, 3.8)	1.0 (0.99. 1.0)	1.6
***Prone bench pull***								
1RM	72.4 ± 16.7	72.8 ± 16.7	-0.4	0.36	-0.02 (-0.39, 0.35)	2.8 (2.1. 3.6)	0.99 (0.98, 1.0)	1.4
L-V relationship	71.3 ± 17.2	70.7 ± 17.1	0.6	0.32	0.04 (-0.34, 0.41)	4.4 (3.2, 5.5)	0.98 (0.97, 0.99)	2.2
MIF	71.5 ± 14.0	72.3 ± 14.0	-0.8	0.09	-0.06 (-0.43, 0.31)	3.5 (2.6. 4.4)	0.98 (0.96. 0.99)	1.8
MRF	72.4 ± 16.0	72.7 ± 16.1	-0.3	0.39	-0.02 (-0.39, 0.35)	2.9 (2.2, 3.7)	0.99 (0.98, 1.0)	1.5

L-V = load-velocity, MIF = maximal isometric force, MRF = maximal repetitions to failure, diff. = difference, ES = effect size, CI = confidence interval, CV = coefficient of variation, ICC = intraclass correlation, SEm = standard error of the measurement. Data for session 1 and 2 are stated in mean ± SD. No significant differences were observed between test session 1 and 2 for any of the tests.

For the prone bench pull, no significant differences between test session1 and 2 were observed for any of the tests, and all between-session differences were trivial (Table 1). CVs ranged from 2.8 to 4.4%, all ICCs were nearly perfect (≥ 0.98), while SEm values were in the range 1.4–2.2 kg.

### Validity

For 1RM prediction of the MIF test results, the following (best fit) equation was used for the bench press: y = 0.0694x – 39.664. Corresponding equation for the prone bench pull was y = 0.0337x – 5.8033, where y = 1RM and x = MIF test result (assessed in Newton). For converting MRF test results to 1RM, the following equation was used for the prone bench pull: y = 1.114x + 1.985z - 26.532, and for the bench press: y = 1.204x + 2.025z – 35.17, where y = 1RM, x = number of repetitions, and z = absolute resistance. The participants managed to perform 31 ± 14 and 23 ± 8 repetitions (mean ± SD) in the bench press and prone bench pull, respectively.

**Fig 2:**
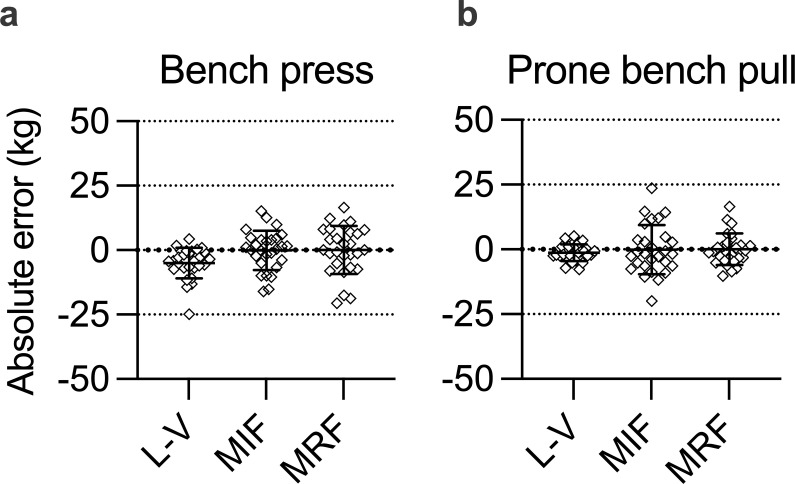
Validity of 1RM estimates across test methods. Panel A denotes bench press, while panel B denotes prone bench pull. The estimates are presented as absolute differences from 1RM. L-V = load-velocity relationship, MIF = maximal isometric force, MRF = maximum repetitions to failure. Values are presented as mean ± SD (*n* = 28).

**Fig 3:**
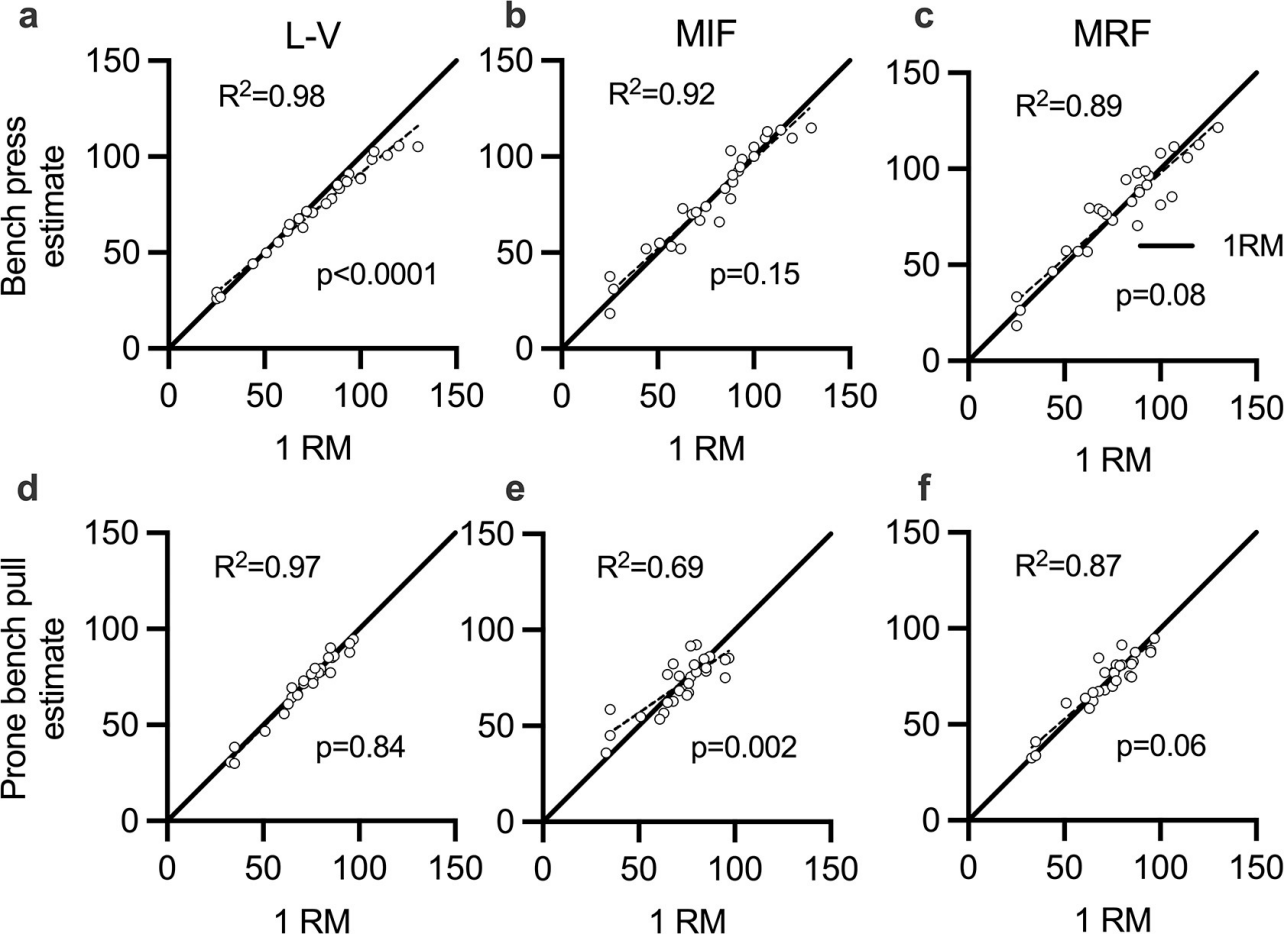
1RM estimates in the bench press (upper panels) and prone bench pull (lower panels) plotted against 1RM with linear regression lines across test methods. Stippled lines represent linear regression slopes, black lines represent slope = 1 for 1RM. P-values denote differences from 1RM slope.

Validity measures for the investigated strength tests are shown in Table 2. Fig 2 shows validity of 1RM estimates across test methods as absolute difference from 1RM, while Fig 3 shows 1RM estimates plotted against 1RM with linear regression lines across test methods. No significant differences were observed among the 1RM predictions and the actual 1RM value, except for L-V vs. 1RM in the bench press (ρ < 0.01). ES for the magnitude of 1RM predictions was trivial for all comparisons, except for L-V vs 1RM in the bench press (small). Here, the average for all subjects was underestimated by 5 kg (Fig 2A). In the prone bench pull, the L-V method underestimated 1RM by 1.3 kg. A plot of the estimated 1RM for all methods against the actual 1RM value showed that the L-V method predicted 1RM with higher accuracy (Fig 3A and 3D). Moreover, superior MAE, SEE and CV values were observed for L-V compared to the other methods (Table 2). All correlations for the bench press and prone bench pull were almost perfect, except for MIF vs. 1RM in the prone bench pull (very high).

**Table 2:** Validity of the 1RM predictions.

*Bench press*	Δ (kg)	*p* for Δ	MAE (kg)	ES (95%CI)	R (95&CI)	CV% (95%CI)	SEE (kg)
L-V vs. 1RM	5.0	<0.01	5.5	0.20 (-0.18, 0.57)	0.99 (0.98, 0.99)	7.8 (5.8, 9.8)	4.2
MIF vs. 1RM	0.1	0.96	5.8	0.00 (-0.37, 0.37)	0.96 (0.92, 0.98)	9.7 (7.1, 12.2)	7.7
MRF vs. 1RM	0.0	0.99	7.2	0.00 (-0.37, 0.37)	0.94 (0.88, 0.97)	11.8 (8.7, 14.8)	9.5
***Prone bench pull***							
L-V vs. 1RM	1.3	0.05	2.8	0.07 (-0.30, 0.44)	0.98 (0.96, 0.99)	4.5 (3.3, 5.6)	3.2
MIF vs. 1RM	0.1	0.96	7.4	0.01 (-0.36, 0.38)	0.83 (0.66, 0.92)	13.2 (9.8, 16.7)	9.7
MRF vs. 1RM	0.0	0.99	4.6	0.00 (-0.37, 0.37)	0.93 (0.86, 0.97)	8.5 (6.2, 10.7)	6.2

L-V = load-***velocity***, MIF = maximal isometric force, MRF = maximal repetitions to failure, Δ = mean difference, MAE = mean absolute error, ES = effect size, R = Pearson’s R correlation, CV = coefficient of variation, SEE = standard error of the estimate, CI = confidence interval.

For the bench press, the change score between sessions 2 and 3 for the direct 1RM assessments were significantly correlated (ρ < 0.05) with predicted 1RM change scores from the L-V relationship method (*r* = 0.69; 95%CI; 0.42, 0.84), MIF (*r* = 0.64; 0.35, 0.82) and MRF (*r* = 0.64; 0.35–0.82), respectively). For the prone bench pull, the change scores for direct 1RM measurements were significantly correlated with predicted 1RM change scores for the L-V relationship (*r* = 0.74; 0.50, 0.87) and MRF (*r* = 0.57; 0.24, 0.77).

## Discussion

This study aimed to explore the validity and reliability of different upper body push and pull tests to determine the 1RM load. The main findings were that trivial differences were observed between test day 1 and 2 for the investigated tests (0.6 to -1.3 kg), except for MIF in the bench press (-3.2 kg; small), while ICCs were practically perfect for all tests. Most CVs and SEm values were in the range 2.3–4.4% and ~1–2 kg, respectively, except for MIF in the bench press (8.3% and 4.2 kg, respectively). Regarding validity, all predictions showed a mean difference of ≤ 1.3 kg (trivial) compared to the actual 1RM value, except for the L-V relationship method, which underestimated the actual 1RM load in the bench press by 5 kg (small). However, smallest deviations in 1RM predictions were observed for the L-V relationship method. Change scores for all the investigated tests were significantly correlated with change scores in actual 1RM values (high to very high), except for MIF in the prone bench pull.

The L-V relationship method applied in this study exhibited reliability values on par with direct 1RM measurements, confirming observations of former investigations [15–18, 25, 26]. The ability of the L-V relationship method to accurately predict 1RM in both bench press and prone bench pull exercises is also in line with previous studies [15, 18, 27–29]. However, the under-estimations revealed here are more pronounced than those previously reported, particularly for the bench press exercise. Garcìa-Ramos et al. [30] also reported that their generalized group equations systematically underestimated the actual 1RM load when predicted from the concentric action but overestimated this load when predicted from the eccentric-concentric action. The plots in Fig 2 revealed that 1RM underestimations for the L-V relationship method in this study were most prominent among the strongest participants. This is likely because the strongest individuals were not tested sufficiently close to their maximal strength capacity. Our standardized protocol was probably not suited for the strongest participants, as heavier loads should have been applied. However, the slope of the L-V estimates was very well aligned with the 1RM slope for athletes in the mid-to-lower part of the 1RM spectrum. Ruf et al. [26] also observed that the prediction models were more accurate when heavier loads (up to 90% of 1RM) were included. Previously published studies have reported acceptable accuracy with only two load increases (e.g., ~40 and ~85% of 1RM), provided that the last resistance load is ≥ 80% of real 1RM [27–29]. Indeed, the two-point method is less time consuming compared to multiple point methods. However, implementing resistance loading close to maximal capacity contradicts the rationale for submaximal testing as the intention is to avoid possible downsides (e.g., fatigue, prolonged recovery time, increased injury risk) associated with heavy loading conditions in subjects with varying training backgrounds.

The predictions of 1RM with the L-V method in this study were based on pre-determined TV group values from pilot testing. Interestingly, the TVs of 0.2 m·s^-1^ for the bench press and 0.50 m·s^-1^ for the prone bench pull were in accordance with García-Ramos et al. and Loturco et al. [16, 18, 30, 31]. Several studies have compared the accuracy of individual vs. group TV values for 1RM predictions, with contradicting findings [27, 30, 32, 33]. The discrepancies can be explained by differences in exercises, age, athlete performance level, and training background across the investigations. Seemingly, the between-participant variation in MVT is practically identical to the within-athlete variation [18, 25, 26, 33]. Since the group-based approach is more time efficient (it does not require each participant to perform 1RM testing in advance to determine individual TV), a group-based TV was chosen for all participants in this study. It should be noted that a very recent approach, the so called optimal minimum velocity threshold method, has shown more accurate 1RM estimates compared to both group-based and individual TVs [34].

The MIF tests showed poorer reliability in comparison with the L-V relationship method. Regarding validity, it was expected that the raw differences in 1RM estimations should be very close to 0 kg, as best fit linear equations derived from the present dataset were used to estimate 1RM from the MIF tests. Of note, these equations may be valid only for the current participants, and caution is therefore warranted when applying them to other populations. However, larger deviation in 1RM predictions in terms of MAE, CV and SEE were observed for MIF compared to the L-V relationship method, and the deviations were more pronounced in the prone bench pull compared to the bench press (Table 2 and Fig 2). Moreover, MIF was the only method not showing a significant change-score relationship with the actual 1RM load in the prone bench pull. Our results are somewhat in contrast to previous studies concluding that MIF is a valid and reliable tool to determine the 1RM load [25, 35, 36]. One possible explanation for these discrepancies may be related to the choice of joint angle. In the present study, the maximal isometric prone bench pull was performed with straight arms, while the corresponding bench press test was performed with an elbow angle of 45°. Bellar et al. [35] applied 90° elbow angle reported during MIF in the bench press and reported acceptable reliability and validity to predict 1RM. However, the participants in this study performed the isometric test in a push-up position while tethered to a load cell anchored to the ground. Lum & Aziz [36] reported that MIF obtained from isometric prone bench pull at both 90 and 120° elbow angles predicted 1RM with equal precision. Moreover, Comfort et al. [37] observed no substantial differences in force production across a wide range of knee and hip joint angles in the mid-thigh pull, even though an individually preferred position showed superior reliability and lowest measurement error.

The MRF test showed reliability values on par with the gold standard 1RM measurements for the bench press and the prone bench pull exercises. As for the MIF tests, it was expected that the raw differences in 1RM predictions should be very close to 0 kg. However, larger deviations in 1RM predictions emerged when comparing to the L-V approach, an observation in line with previous studies [18, 29]. Indeed, resistance loading and number of repetitions during MRF are critical for 1RM prediction accuracy. The heavier the resistance (relative to actual 1RM), the higher prediction accuracy [10, 37]. According to Reynolds et al. [10], no more than 10 repetitions should be used in linear equations to estimate the 1RM value. In the present study, the number of repetitions were in the range 4–50, with the most experienced lifters in the upper end. This large variation may be methodologically problematic, but either heavier or lighter loads relative to bodyweight (i.e., % bodyweight), for both women and men, would probably not have solved the problem for this heterogeneous subset. Moreover, preliminary 1RM testing for more targeted resistance loadings contradicts the main purpose of the MRF method as a gentler and safer test than the traditional time-consuming 1RM measurement. However, it is reasonable to argue that multiple repetitions at lighter loads lead to the same or even higher degrees of fatigue and subsequent need for recovery compared to heavier loads. In this context, the rationale of MRF testing can be questioned. Therefore, caution is required when applying methods based on a high number of repetitions (and light loads) and using general L-V equations to determine the 1RM load across different samples, as these equations may be valid only for the subsets in which they were based on.

Some study limitations should be acknowledged. Firstly, all tests in this study were performed in a Smith Machine. That is, the bar patch was fixed throughout the movements. Free-weight exercises possess other kinetic and kinematic movement characteristics, as there is typically more movement in the sagittal plane and higher range of motion when using free-weights [38]. Although caution is warranted when generalizing the present findings to free-weight movements, Loturco et al. [16] reported that both free-weights and Smith Machine could predict 1RM bench press with similar accuracy in top-level athletes. Moreover, our reliability analyses (Table 1) revealed a slight trend towards better test results in session 2, indicating insufficient familiarization. However, the effect magnitudes of these differences were mainly trivial, except for MIF in bench press where a small effect (3.2 kg) was observed. With more familiarization in advance, these reliability values would likely have improved. However, because this data collection was performed during a Covid-19 period with restrictions/partial lockdown and limited time window, we prioritized sample size (statistical power) over familiarization. Finally, the differences in strength training background among the participant must also be acknowledged as a potential study limitation, and caution is warranted when generalizing the current findings to more homogeneous subsets.

## Conclusion

All tests examined in this study, with exception of the MIF, were able to track training-related changes, indicating good validity. Although our L-V relationship approach slightly underestimated the actual 1RM load, this method exhibited the smallest deviations in 1RM (and relative load) predictions. Given the trivial-to small differences between predicted and actual 1RM values, and the high variability associated with individual predictions for each approach, the 1RM prediction methods analyzed herein cannot be used interchangeably. However, indirect and submaximal strength tests are always good alternatives for assessing maximum strength capacity, especially when traditional 1RM measurements are not feasible. In such cases, the L-V relationship approach is particularly recommended since this method exhibits the highest degree of precision to determine the 1RM load.

## Supporting information

S1 Data (XLSX)
